# Partial suppression of a strongly expressed tonoplast sucrose transporter affects water use and carbon partitioning in *Populus*

**DOI:** 10.1186/1753-6561-5-S7-P111

**Published:** 2011-09-13

**Authors:** Scott Harding, Christopher Frost, Raja Payyavula, Kate HC Tay, Chung-Jui Tsai

**Affiliations:** 1 University of Georgia, USA

## 

Sucrose export from source organs, and its subsequent distribution among differentiating organs in wood-forming stems and elsewhere depends on the activity of sucrose transporters (SUTs). There are no comprehensive reports on SUT function in temperate tree species valued for their lignocellulosic biomass. To begin to address this gap, the SUT gene family was characterized and functionally analyzed in transgenic *P. tremula x alba*. The *Populus* SUT family features the three major groups characteristic of other dicots. In general, functionally distinct SUTs fall into different phylogenetic groups. Group-1 *PtaSUT3* transcripts localize to leaf vascular traces and stem developing xylem; Group-4 *PtaSUT4* to leaf spongy mesophyll, stem developing xylem, cambium and phloem; Group-2 *PtaSUT5/6* to all leaf cells, stem developing xylem and phloem fibers. The SUT4 ortholog of *Populus* differs from that of other model plants in encoding a vacuolar transporter that is unusually well expressed in source leaves compared to Group-1 and 2 SUT genes. SUT4-RNAi transgenic plants demonstrated a shift of biomass allocation from stem to leaf in both nitrogen (N)-replete and N-limited plants. In those plants, sucrose exhibited a complex pattern of hyper-accumulation in exporting leaves and vascular tissues of the stem, and decreased accrual in the shoot tip and sink leaves. RNAi silencing of SUT4 reduced water uptake during drought simulation without significantly affecting overall shoot biomass accumulation.

